# The construct validity of the health utilities index mark 3 in assessing health status in lung transplantation

**DOI:** 10.1186/1477-7525-8-110

**Published:** 2010-09-28

**Authors:** Maria-Jose Santana, David Feeny, Sunita Ghosh, Ronald G Nador, Justin Weinkauf, Kathleen Jackson, Marianne Schafenacker, Dalyce Zuk, Grace Hubert, Dale Lien

**Affiliations:** 1 Lung Transplant Program. 2E4.31 Walter C. Mackenzie Health Sciences Centre. University of Alberta Hospital. Edmonton. T6G2B7, Alberta, Canada; 2 The Center for Health Research. Kaiser Permanente Northwest, 3800 N. Interstate Avenue, Portland 97227-1110, OR, USA; 3 Experimental oncology. Cross Cancer Institute. 11560 University Avenue. Edmonton, T6G 1Z2, Alberta, Canada; 4 Lung Transplant Program. Clinical Sciences Building. University of Alberta Hospital. Edmonton. T6G2B7, Alberta, Canada; 5 2C2, Walter C. Mackenzie Health Sciences Centre. University of Alberta Hospital. Edmonton. T6G2B7, Alberta, Canada; 6 Lung Transplant Program. 5D1.16 WMC. University of Alberta Hospital. Edmonton. T6G2B7, Alberta, Canada

## Abstract

**Purpose:**

To assess the cross-sectional construct validity of the Health Utilities Index Mark 3 (HUI3) in lung transplantation.

**Methods:**

Two hundred and thirteen patients (103 pre-transplant and 110 post-transplant) with mean age 53 years old (SD 13) were recruited during a randomized controlled clinical trial at the out-patient clinic in a tertiary institution. At baseline, patients self-completed measures that included the HUI3, EuroQol EQ-5D, Hospital Anxiety and Depression Scale (HADS) and socio-demographic questionnaire. Six-minute walk test scores and forced expiratory volume in 1 second data were collected from patient's medical records. *A priori *hypotheses were formulated by members of the transplant team about the expected degree of association between the measures. Correlation coefficients of < 0.1 were considered as negligible, 0.1 to < 0.3 as small, 0.3 to < 0.5 as medium, and ≥0.5 as large.

**Results:**

Of the ninety predictions made, forty three were correct but in 31 the correlation was slightly lower than predicted and in 7 the correlations were much higher than predicted. In 48% of the cases, predicted and observed associations were in agreement. Predictions of associations were off by one category in 42% of the cases; in 10% of the cases the predictions were off by two categories.

**Conclusions:**

This is the first study providing evidence of cross-sectional construct validity of HUI3 in lung transplantation. Results indicate that the HUI3 was able to capture the burden of lung disease before transplantation and that post-transplant patients enjoyed higher health-related quality of life than pre-transplant patients.

## Background

The major end-points in lung transplantation are survival and health-related quality of life (HRQL). HRQL assessments are important for understanding the impact of treatment on patients, including physical functioning and emotional well-being. Recent studies shown that after transplantation the most significant improvements were reported in physical and social functioning, and overall HRQL [1-10], whereas psychological problems seemed to be prevalent after the transplant [2,10]. In lung transplantation, the most commonly used measures are health profiles, like the SF-36 [11]. Health profiles do not incorporate values/preference information which requires such data for the estimation of quality-adjusted life years (QALY). As a result health profiles measures are not suitable for use in economic evaluations comparing the cost-effectiveness of different treatments and interventions.

In lung transplantation, the determination of relative benefits and costs of different treatments and interventions are of importance to clinical care optimization. Therefore, recently studies have incorporated preference-based measures [6,10,12,13]. There are two types of preference-based measures: direct and multi-attribute. Direct measures, visual analog scales (VAS), time trade-off (TTO) and standard gamble (SG) assess the preference for a health state and are suitable for specific purposes allowing the researcher to incorporate items that are more relevant to a particular population. Multi-attribute preference measures, such as Health Utilities Index Mark 2 (HUI2) [14] and Mark 3 (HUI3) [15], EuroQol (EQ-5D) [16], SF-6D [17] and Quality of Wellbeing questionnaire (QWB) [18], describe the health status of a subject using a multi-attribute classification system and use a scoring system to value health status.

Compared with other multi-attribute preference measures, the HUI3 was selected for several reasons. First, the SF-6D [17] has floor effects. The QWB [18] scale is lengthy, increasing the burden to patients. The HUI3 has more breadth and depth (HUI3 includes 8 attributes with 5 to 6 levels in each) than the EQ-5D [16] (includes 5 attributes with 3 levels in each) providing more detailed information on the patient's health status for clinicians. The EQ-5D has ceiling-effect problems and often misses health states with mild burdens. Lung transplant recipients are fairly close to population norms and typically experience states with mild burdens. The EQ-5D has the potential to misinterpret health status because it does not include levels for mild problems, as seen in the gap in the scores between 0.88 and 1.00 (perfect health). Thus, EQ-5D may identify a patient as experiencing perfect health when in reality that patient is experiencing a health state with a mild burden.

HUI3 provides detailed information about patient's health status by including an overall score and single-attribute utility scores. The HUI3 includes eight attributes (vision, hearing, speech, ambulation, dexterity, cognition, emotion, pain and discomfort) with five or six levels for each attribute [14,15,19]. The single-attribute utility scores convey information about the degree of disability in each attribute. Furthermore, HUI3 [15] is useful because describes a great number of health states, and captures the severity of the disease and burden of side-effects associated with drugs and other treatments, and the burdens associated with comorbidities. For instance, symptoms such as fatigue and breathing limitations will limit ambulation. Also, changes in emotional states due to some treatments may be present in some patients and captured by HUI3 emotion. Pain will limit patients' ambulation and health status.

The HUI3 has been used in population health surveys in Canada since 1990 [20]. The validity of the HUI3 has been demonstrated for various diseases as well as the general population [21-32]. Recently, the HUI3 has been used in lung transplantation [10,33]. Santana et al [10] using the HUI3 followed prospectively 43 pre-transplant patients after six months post-transplantation. In this study the HUI3 was able to detect improvement after transplant. However, the present study is the first to add evidence on the cross-sectional construct validity of the HUI3 in lung transplantation. We examined convergent validity, divergent validity and the known-groups approach.

Construct validity is an important component in the evaluation of the performance of HRQL measures. The assessment of construct validity is an on-going exercise that requires the accumulation of evidence about the performance of a measure in different settings. One way to assess construct validity is the extent to which a particular measure relates to other measures in a way that is consistent with theoretically derived hypotheses related to the concepts that are being measured. Thus, measures are valid when they measure what they are supposed to measure [34,35]. And measures are responsive when they are able to capture meaningful change over time. Convergent validity considers the direction and degree of association that one expects to observe among measures of the same or a similar construct. For example ambulation scores would be highly related to and systematically vary with six-minute walk test scores. In contrast for discriminative validity one examines the degree of association when little or no association among the constructs is expected. For instance, ambulation scores are not expected to be highly related to patient's marital status. Known-groups comparison is another approach for assessing construct validity. One anticipates that specific groups of patients will score differently from others, thus the measure should be sensitive to these differences. On the basis of independent evidence based on clinical measures, we would expect that HUI3 would discriminate between pre- and post-transplant patients.

## Methods

### Patients and Procedure

The patient sample included pre-lung transplant (subjects who were included on the waiting list and were being seen at the out-patient clinic) and post-lung transplant subjects. Patients were excluded if they were younger than 18 years of age, diagnosed as being cognitively impaired, or unable to complete questionnaires in English.

The main study was a randomized controlled clinical trial that assessed the effect of using HRQL measures in routine clinical care of lung transplant patients [33]. The study was conducted at the lung transplant out-patient clinic, at the University of Alberta Hospital, Edmonton. The out-patient lung transplant team consisted of three physicians, two nurses, one pharmacist, and one dietician. Ethics approval was obtained from the Health Research Ethics Panel B, file # 101004, University of Alberta.

Baseline data was collected at the first patient visit once patient consent had been obtained. At baseline, patients self-completed a battery of paper-and-pencil questionnaires: socio-demographic, Hospital and Anxiety Depression Scale (HADS), Health Utilities Index Mark 3 (HUI3), and EQ-5D. Pulmonary function test was conducted at the pulmonary laboratory and the six-minute walk test (6MWT) was performed at the Physiotherapy Department.

### Health Status and Health-related Quality of Life Measures

#### Health Utilities Index Mark 3, HUI3

The 15-item HUI self-assessment self-complete one-week recall questionnaire was used in the study. The levels range from severe disability (e.g., so unhappy that life was not worthwhile) to no disability (e.g., happy and interested in life) [15,19]. HUI3 describes a total of 972,000 unique health states. An individual health status is described by an eight-element vector, with one level for each attribute. The HUI3 scoring function is a multiplicative multi-attribute that was developed based on community preferences obtained from a random sample of the Canadian population [15]. The HUI3 single-attribute utility scores (SAUS) are on a scale in which the score for most highly impaired level is 0.00 and the score for normal is 1.00. HUI3 overall scores are on a scale in which the all-worst HUI3 state (every attribute is at its highest level of disability) has a score of -0.36 (negative scores reflect health states considered by to be worse than being dead), dead is 0.00 and perfect health is 1.00. Changes of 0.03 or more in overall HUI scores and 0.05 or more in single-attribute scores are considered clinically important [19].

#### Euroqol, EQ-5D

EQ-5D, a brief generic preference-based measure that consists of two components: a 100-point visual analog scale (VAS) and a descriptive system [16]. The 20 cm VAS ranges from 0 (worst imaginable health) to 100 (best imaginable health). Patients are asked to rate their own health that day by drawing a line from a box to a point on the VAS. The descriptive or self-classification system contains five attributes (mobility, self-care, usual activities, pain or discomfort, and anxiety or depression) with three levels per attribute ("no problem", "some problems" and "extreme problems"). The EQ-5D classification system generates 243 possible health states [16]. Using the US scoring function EQ-5D index scores range from -0.11 (all-worst health state, worse than dead), to 0.00 (dead) to 1.00 (perfect health) [36]. The scoring function was estimated using time trade off scores from a representative sample of the community-dwelling US population. Changes of 0.10 or more in EQ-5D index are considered clinically important.

#### The Hospital Anxiety and Depression Scale (HADS)

Mental health was assessed using the HADS [37]. HADS is a self-complete mental health measure. The scale consists of 14 items, 7 of which assess anxiety and 7 which assess depression. Each item is on a four point scale and the scores are added to give a total ranging from 0 to 21 for anxiety and 0 to 21 for depression. Higher scores indicate higher severity of anxiety or depression. A cut-point of 8 or 9 indicates mild burden for the two scales; 11 or 12 indicates severe [37]. HADS uses a one week recall period. HADS has been used to measure anxiety and depression in community screening and clinical research.

#### Patient sociodemographic characteristics

At the first study visit (baseline assessment) the patients completed a brief sociodemographic questionnaire. The purpose was to provide a description of sociodemographic characteristics of this patient population. Items included age, gender, level of education, and employment status.

#### Chronic conditions

Patients were asked whether they have been diagnosed with any of the following conditions: arthritis or rheumatism, high blood pressure, asthma, chronic bronchitis or emphysema, diabetes, epilepsy, effects on stroke (paralysis or speech problems), paralysis, partial or complete, other than the effects of a stroke, urinary incontinence, difficulty controlling bowels, Alzheimer disease or any other dementia, osteoporosis or brittle bones, cataracts, glaucoma, stomach or intestinal ulcers, kidney failure or disease, Crohn disease or colitis(bowel disorder), thyroid condition, developmental delay, schizophrenia, depression, psychosis or other mental illness, cancer. The number of chronic conditions was calculated for each patient.

#### Pulmonary Function

Patients' medical records were reviewed to obtain the 6-minute walk test (6MWT) scores and the forced expiratory volume, FEV_1 _percentage predicted, closest in time to the date at which the patient enrolled in the study. The cut-off point for FEV1 %predicted was ± 3 days of when HRQL was assessed; for the 6MWT the cut-off was ± 5 days.

### Formulation of *a priori *hypotheses

Seven out of the ten authors independently indicated the direction and degree of expected association among the measures in order to assess convergent and discriminant validity. Each author specified 90 *a priori *hypotheses, of which 52 tested convergent and 38 discriminant validity. *A priori *hypotheses were specified by members of a multi-disciplinary team of clinicians that included pulmunologists, nurses, a pharmacist and a dietitian. All these predictions were compiled and a consensus was reached for each of the 90 hypotheses by endorsement of a proposed consensus set of hypotheses. To classify the degree of association, we used the scheme provided by Cohen (1988) [38] negligible (< 0.1), small (0.1 to < 0.3), medium (or moderate) (0.3 to < 0.5), large (> 0.5).

To test convergent validity, we expected that patients with a higher ambulation score to walk further in the 6MWT and to display a higher FEV1%pred score. Also, HUI3 pain that covers activity disruption due to pain was expected to be moderately and negatively correlated with 6MWT, as patients experiencing pain and discomfort would have difficulty walking. Furthermore, HUI3 emotion focuses on happiness versus depression and was expected to be largely correlated to HADS depression score.

Discriminative validity was demonstrated through testing *a priori *hypotheses in situations in which we expected to find a negligible correlation between the measures. For instance, because vision is not expected to be related to the pulmonary function, we expected HUI3 vision to be negligibly correlated with FEV1%pred. Similarly, marital status was expected to be negligibly correlated with HUI3 cognition.

To assess the known-groups comparisons, we expected that pre-transplant patients with symptoms such as fatigue and breathing limitations would experience limited ambulation, thus displaying lower HUI3 ambulation than post-transplant subjects. Also, pre-transplant patients (waiting for transplant) would display lower HUI3 pain scores (more pain) than post-transplant patients. At end-stage lung disease some patients (pulmonary fibrosis and arterial hypertension) suffer pleureitic chest pain. Other pre-transplant patients (chronic obstructive pulmonary disease) use the accessory breathing muscles which leads to back and thoraxic cage pain. Also it was expected that post-transplant subjects would report higher overall HUI3 than pre-transplant patients.

### Statistical analyses

The statistical analyses were conducted by one of the authors who was not involved in the formulation of the *a priori *hypotheses. Pearson correlations were estimated for continuous variables; Spearman's Rho test was used for categorical variables, and unweighted kappa was calculated to assess agreement between the predicted and observed degrees of association. Agreement is interpreted following the scheme proposed by Altman [39] < 0.20, poor; 0.21-0.40, fair; 0.41-0.60, moderate; 0.61-0.80, good; 0.81-1.00, very good. Student's t-tests were performed to assess the known-group comparisons.

The statistical analyses were computed using SPSS version 15.0 [40].

## Results

The study was carried out between July 2005 and April 2007. During this period, 216 patients were invited to participate. Three pre-transplant patients refused. Out of the 213 enrolled patients, 103 were pre-transplant (52% female) and 110 were post-transplant patients (46% female). Table 1 presents the baseline demographic and clinical characteristics for the 213 patients. Patients had a mean age of 53 years with a range from 18 to 73 years. Most of the patients had finished high school and were on disability. Thirty one percent of the pre-transplant patients rated their general health as poor versus four percent in the post-transplant group. Similarly, fourteen percent of the pre-transplant patients rated their general health as good versus thirty eight percent in the post-transplant group. The most common chronic conditions were osteoporosis, arthritis, hypertension and diabetes. The most common underlying diagnoses were chronic obstructive pulmonary disease (COPD) and idiopathic pulmonary fibrosis (IPF). These results are consistent with the distribution of causes for lung transplantation by country [41]. At enrollment in the study the mean time waiting for transplant was 81 weeks (range from 1 to 158 weeks) for the pre-transplant group and the mean time since transplant was 136 weeks (range 3 to 960 weeks) for the post-transplant group.

**Table 1:** Demographic and clinical characteristics of the patients at baseline

	Pre-transplant N = 103	Post-transplant N = 110
Mean Age (SD)	54 (12.55)	53 (12.93)

Gender (%)		
Female	55	44
Male	45	56

Race/Ethnicity (%)		
White	98	92
American Indian	2	3
East Indian	0	2
Asian	0	1
Black	0	1

Marital Status (%)		
Married	47	53
Single	50	50
Divorced	68	32
Other	46	55

Education (%)		
High school	46	54
College	60	40
University	32	68

Employment (%)		
Working	15	18
Unemployed	16	13
Retired	21	22
Disability	48	47

General Health (%)		
Excellent	1	6
Very Good	14	38
Good	22	38
Fair	32	14
Poor	31	4

Chronic Conditions (%)		
Arthritis	20	15
Osteoporosis	24	33
Hypertension	26	31
Diabetes	11	18
Other	9	3

Co-morbidities (%)		
Chronic Obstructive Pulmonary Disease	43	41
Pulmonary Fibrosis	29	27
Pulmonary Arterial Hypertension Cystic Fibrosis	10 15	11 19
Other	3	2

Mean Number of Chronic conditions (SD)	2.00 (1.74)	1.48 (1.56)

Mean Six Minute Walk test, in meters (SD)	357 (134)	548 (155)

Mean FEV1% pred* (SD)	39.20 (21.63)	67.10 (25.19)

Mean time since transplantation (weeks)		136 (range 3-960)

SD = Standard Deviation; *FEV1%pred = Predicted Forced Expiratory Volume in 1 second

The age-matched (matched to the age distribution of the patients) Canadian HUI3 norm for men is 0.89 and 0.90 for women, both indicating mild disability [10]. The mean HUI3 overall score of 0.63 for the patients indicates moderate to severe disability (see Table 2). Overall scores ranged from 0.001 to 1.00. HUI3 pain and HUI3 ambulation (0.80 and 0.78, respectively) were the most severely affected attributes (see Table 2). The number of chronic conditions ranged from 0 to 10, consistent with the severity captured by the overall HUI3 score (see Table 2). The functional status of the patients assessed by the mean 6MWT was moderate [42] 448 meters (SD 173 meters). Also, a mean percentage of predicted FEV1 of 54 (SD 27.4) showed moderate [43] chronic airflow impairment. These results are consistent with the severity captured by the overall HUI3 score (see Table 2).

**Table 2:** Description of patients HRQL

HRQL Measures	Pre-transplant Mean ± SD	Post-transplant Mean ± SD	Difference between mean scores for post- and -pre-transplant patients
HUI3 vision	0.94 ± 0.12	0.92 ± 0.12	- 0.02

HUI3 hearing	0.94 ± 0.20	0.96 ± 0.17	0.02

HUI3 speech	0.99 ± 0.07	0.97 ± 0.15	0.02

HUI3 ambulation	0.66 ± 0.28	0.89 ± 0.19	0.23*^†^

HUI3 dexterity	0.99 ± 0.02	0.97 ± 0.10	0.02*

HUI3 emotion	0.93 ± 0.10	0.94 ± 0.12	0.01

HUI3 cognition	0.93 ± 0.10	0.94 ± 0.12	0.01

HUI3 pain	0.76 ± 0.26	0.84 ± 0.17	0.08*^†^

HUI3 overall	0.56 ± 0.26	0.69 ± 0.25	0.13*^†^

EQ-5D index	0.71 ± 0.17	0.81 ± 0.15	0.10*^†^

HADS anxiety	6.83 ± 3.44	5.42 ± 3.57	1.41*

HADS depression	5.82 ± 2.84	3.34 ± 3.30	2.48*

* Statistically significant (p < 0.05); †clinically important difference

Using the known-group approach, we expected the pre-transplant patients to have lower overall HUI3, and lower HUI3 ambulation and HUI3 pain scores than post-transplant patients. Differences between pre- and post-transplant in overall, ambulation and pain were statistically significant and clinically important (see Table 2).

The observed correlations are reported in Table 3. Twelve out of the 52 hypotheses testing convergent validity and 5 out of the 38 testing discriminant validity were not confirmed. Of the ninety predictions made, forty three were correct but in 31 the correlation was slightly lower than predicted and in 7 was much higher than predicted. The correlation between HUI3 overall score and EQ-5D index was large (p = 0.001). HUI3 ambulation and HUI3 pain correlated moderately with EQ-5D index (p = 0.001). Correlations between EQ-5D and HUI3 vision, hearing, speech, dexterity and cognition were negligible (p > 0.05). HUI3 emotion correlated moderately with HADS anxiety (p = 0.001) and HADS depression (p = 0.001). Correlation between HUI3 ambulation and 6MWT was large (p = 0.001). Also, there was a small correlation between HUI3 pain and the 6MWT (p = 0.002). As expected, marital status and HUI3 ambulation did not correlate (p = 0.31). Also, HUI3 dexterity did not correlate with FEV1 (p = 0.36).

**Table 3:** Observed correlations

	EQ-5D index	HADS anxiety	HADS depression	6MWT	FEV1% pred	NCC	Age	Gender	Marital Status	Transplant Status
HUI3 overall	**0.50**	**-0.43**	**-0.55**	**0.35**	**0.25**	**-0.20**	**-0.13**	**0.15**	**0.03**	**0.25**

HUI3 vision	0.04*	-0.06*	-0.06*	0.01*	0.02*	-0.02*	**0.20**	0.12*	0.01*	0.05*

HUI3 heari**n**g	0.08*	-0.11*	**-0.20**	0.08*	0.11*	0.07*	**0.15**	0.02*	0.00*	0.03*

HUI3 spee**c**h	0.02*	-0.24	-0.13*	0.05*	0.02*	-0.01*	0.01	0.00*	0.02*	0.07*

HUI3 ambulation	**0.40**	**-0.24**	**-0.50**	**0.59**	**0.36**	**-0.19**	-0.15*	0.16*	0.00*	**0.43**

HUI3 dexterity	0.02*	0.11*	0.05*	0.05*	0.06*	0.13	-0.10*	0.03*	0.05*	0.17

HUI3 emotion	**0.12**	**-0.40**	**-0.43**	-0.08*	0.08*	-0.01*	0.03*	0.02*	0.06*	0.01*

HUI3 cognition	0.08*	**-0.25**	**-0.19**	-0.02*	0.01*	-0.08*	0.12*	0.11*	0.08*	0.08*

HUI3 pain	**0.44**	**-0.23**	**-0.26**	**0.17**	0.09*	-0.10*	0.03*	0.02*	0.03*	**0.17**

6MWT: Six-minute Walk test; FEV1: Percentage predicted Forced Expiratory Volume in 1 second; NCC: Number of Chronic Conditions;

Transplant Status: pre- or post-transplant

* Non-significant correlations

**Bold**: test of convergent validity; unbold: test for discriminant validity

The accuracy of the *a priori *hypotheses is reported in Table 4. The degree of agreement between *a priori *hypotheses and observed correlations is reported in Table 5. In 48% of the cases (43 out of 90) the predictions were correct. In 42% of the cases predictions were off by one category. *A priori *predictions were off by two categories in 10% of the cases. The chance-corrected agreement measured by unweighted Kappa statistics was 0.25 (p = 0.0001), indicating fair chance-corrected agreement between the observed and the predicted associations.

**Table 4:** *A priori *and observed associations

	EQ-5D index	HADS anxiety	HADS depression	6MWT	FEV1% pred	NCC	Age	Gender	Marital Status	Transplant Status
HUI3 overall	**L**	**M**	*M/L*	**M**	***M/S***	L/S	***M/S***	**S**	***S/N***	**M**

HUI3 vision	M/N	M/N	***S/N***	***S/N***	**N**	**N**	***M/S***	*N/S*	**N**	**N**

HUI3 hearing	M/N	***M/S***	***M/S***	**N**	*N/S*	**N**	***M/S***	**N**	**N**	**N**

HUI3 speech	***S/N***	*N/S*	**S**	**N**	**N**	**N**	**N**	**N**	**N**	**N**

HUI3 ambulation	***L/M***	***M/S***	**L**	**L**	***L/M***	***M/S***	***M/S***	*N/S*	**N**	***L/M***

HUI3 dexterity	M/N	***M/S***	M/N	**N**	**N**	*N/S*	**S**	***N***	**N**	*N/S*

HUI3 emotion	L/S	***L/M***	***L/M***	***S/N***	M/N	***S/N***	***S/N***	**N**	**N**	**N**

HUI3 cognition	**N**	***M/S***	***M/S***	**N**	**N**	**N**	**S**	**S**	**N**	**N**

HUI3 pain	***L/M***	***M/S***	***M/S***	***M/S***	M/N	***M/S***	***S/N***	**N**	**N**	***M/S***

6MWT: Six-minute Walk test; FEV1% pred: Percentage predicted Forced Expiratory Volume in 1 second; NCC: Number of Chronic Conditions

N = negligible degree of association, correlation < 0.1; S = Small degree of association, correlation 0.1 to < 0.30; M = medium degree of association, correlation 0.30 to < 0.5; L = large degree of association, correlation ≥ 0.5

**Bold **= a perfect match between a priori and observed; *italics *= a difference of one category in which a priori < observed;

***bold italic ***= a difference of one category in which a priori > observed; underline = a difference of two categories in which a priori < observed; double underline = a difference of two category in which a priori > observed;

**Table 5:** Accuracy of *a priori *predictions

	N = 90	%
Exact	43	48

Off by 1 category	38	42

a priori > observed	31	
a priori < observed	7	

Off by 2 category	9	10

a priori > observed	9	
a priori < observed	0	

## Discussion

This study is the first to explore the cross-sectional construct validity of the HUI3 in lung transplantation. In particular, 90 hypotheses concerning the associations between HUI3 single attribute utility scores and overall HUI3 utility scores and various measures of health status such as pulmonary function (FEV1% predicted) and the six-minute walk test were examined. Of the 90 hypotheses 43 predictions were exact, 40 were slightly lower than predicted and 7 were slighted higher than predicted. Overall, the results provide evidence supporting the cross-sectional construct validity of HUI3 in lung transplantation.

Our results are similar to results in previous studies investigating construct validity [22,44,45]. Two of the studies included asthmatic children and their caregivers, reporting success rates (% of *a priori *hypotheses that were confirmed) of 55.6% and 50%, respectively. The third study included high-risk primary-care patients and reported a success rate of 50%. However, in 2004 Blanchard et al [24] conducted a construct validity study in patients undergoing elective total hip arthroplasty, reporting a success rate of 75%.

Because the HUI3 and the EQ-5D belong to the same group of measures, clinicians expected the correlations between the HUI3 single attributes scores and the EQ-5D to be higher. Clinicians overestimated the correlations between the EQ-5D and the HUI3 in most of the attributes except for HUI3 cognition. However the correlation between the overall HUI3 and EQ-5D scores was large and the prediction was confirmed. A possible explanation for the pattern of results is that the EQ-5D is a cruder measure than the HUI3. HUI3 includes eight attributes with five or six levels each whereas EQ-5D includes four attributes with three levels each. This difference in depth and breadth between the measures allows the HUI3 to provide more descriptive power for highly impaired states. Luo et al [22,25] noted that EQ-5D was not able to differentiate health status at higher levels of functioning.

The correlation between HUI3 emotion and the HADS anxiety and depression scores was medium. The team expected a higher degree of association for both. The prediction was off by one category. Asakawa et al [30] assessed the construct validity of the HUI3 in Alzheimer disease, arthritis and cataracts. The authors expected a higher degree of association between HUI3 emotion and emotional problems associated to arthritis and cataracts. A possible explanation for our findings is that the HUI3 is a generic measure that focuses on happiness versus depression whereas HADS depression scale is based on anhedonia or the state of reduced ability to experience pleasure [37].

The degree of association expected by clinicians between 6MWT and HUI3 ambulation was correct. However, clinicians were expecting to find a higher degree of association between FEV1% predicted and HUI3 ambulation. The prediction was off by one category. Past studies have addressed the discrepancy in the correlation between FEV1% predicted and HRQL measures [42,46,47]. Poor association between clinical parameters and HRQL scores may be explained by the fact that objectively measurement doesn't reflect patients' perceptions, suggesting that HRQL information is necessary to complement patients' clinical care.

Clinicians were expecting to find a higher correlation between age and cognition. It would be interesting in future studies to examine the degree of association between age and HUI3 cognition in different clinical and age groups. It could be that in this group the major determinants of cognitive status are co-morbidities and degree of severity of their lung disease and other chronic conditions, rather than the age of the patient.

Clinicians' expectations about the degree of association between HUI3 scores and transplant status were confirmed for six out of nine predictions. Predictions for HUI3 ambulation and HUI3 pain exceeded the observed correlation slightly. A possible explanation for the overestimation may be due to the high number (n = 67) of patients who had been transplanted more than a year before enrolling in the study.

When patients were stratified by transplant status (pre- and post-transplant) to examine known-group validity, pre-transplant patients reported lower mean overall HUI3 (0.56) than post-transplant (0.69) patients. The difference was statistically significant (p = 0.005) and clinically important (see Table 2). As expected, HUI3 ambulation and pain were the most affected attributes before transplantation and were much higher in the post-transplant group. The differences were statistically significant (HUI3 ambulation, p = 0.01; HUI3 pain, p = 0.02) and clinically important (see Table 2). The present study corroborated the finding in a previous study [10] confirming that HUI3 ambulation and HUI3 pain were the most affected attributes before transplantation and that overall HUI3 scores were higher in post-transplant patients.

In this study, most of the predictions were confirmed. Over-prediction of the degree of association by one category was more frequent than under-prediction by one category. This pattern was also seen in a study conducted by Feeny et al 2009 [32]. Feeny et al. noted that the success in predicting the degree of associations depends on the validity of the measures used in the study, usefulness of the underlying theory used to derive the hypotheses and knowledge of the measures and study subjects by those who formulate the *a priori *predictions.

In the context of this study, the clinicians who formulated the *a priori *predictions were highly familiar with lung transplantation patients in general and the characteristics of the patients enrolled in the study in particular. These experienced clinicians were also very familiar with standard clinical measures such as the 6MWT and the FEV1% predicted. Many of the clinicians involved in the study were actively using HUI3 in the management of these patients so probably were knowledgeable about that measure, although not knowledgeable about the EQ-5D. The clinicians while knowledgeable about mental health issues were probably not very familiar with the HADS. As noted above the success in confirming *a priori *predictions in this study is consistent with the success rates noted in a number of previous studies. The nature of the theory used to inform *a priori *predictions in this study was for the most part implicit and based on intuitive clinical reasoning and experience. It is possible that the use of a more rigorous and explicit underlying theory would have improved the success rate in predicting the observed degree of associations.

The increasing demands of lung transplantation on health care systems have stimulated much interest in the cost effectiveness of health care interventions in this patient population. Lung transplantation is effective but expensive technology, having a valid utility measure that allow for cost-effectiveness comparison is important. In this study, HUI3 shown to be valid and able to capture both the burden of lung disease before transplantation and the higher levels of health status and HRQL enjoyed by patients after transplantation. Further cost-effectiveness analyses using HUI3 is warranted.

There are a number of study limitations to consider when interpreting these findings. First, patients with cognitive problems and non-English speakers were excluded, limiting generalizability. Secondly, most of the participants were White and recruited at a tertiary-care institution therefore results may not be generalizable to other settings. However, the underlying distribution of causes for lung failure is similar to most cohorts seen internationally. Furthermore, the *a priori *hypotheses were performed at one point in time, at baseline. Because this is the first study to explore the construct validity of the HUI3 in lung transplantation, replication of the study is warranted in future studies. Although responsiveness of the HUI3 has been previously assessed [48,49] the present study did not explore responsiveness of the HUI3 in lung transplantation. A further investigation of the longitudinal construct validity of the HUI3 in lung transplantation is warranted.

## Conclusion

This is the first study that provides evidence of the cross-sectional construct validity of HUI3 in lung transplantation. Results indicate that the HUI3 was able to capture both the burden of lung disease before transplantation and the higher levels of health status and HRQL enjoyed by patients after transplantation.

## Abbreviations

HRQL: Health-related Quality of Life; HUI3: Health Utilities Index Mark 3; EQ-5D: EuroQol health utility instrument; HADS: Hospital Anxiety and Depression Scale; 6MWT: 6-minute walk test scores; FEV_1 _% predicted: Forced expiratory volume in 1 second

## Competing interests

It should be noted that David Feeny has a proprietary interest in Health Utilities Incorporated; Dundas, Ontario, Canada. HUInc distributes copyrighted Health Utilities Index (HUI) materials and provides methodological advice on the use of the HUI. None of the other authors declared any conflict of interest.

## Authors' contributions

All the authors have made substantive intellectual contributions to the study and have given final approval of the version to be published. MJS have made substantial contributions to conception and design, or acquisition of data, or analysis and interpretation of data, and drafting the manuscript. DF made substantial contributions to drafting the manuscript and revising it critically for important intellectual content. SG performed the statistical analysis. All the other authors participated in the formulation of the *a priori *hypotheses and contributed to the drafting of the manuscript.

## Authors' information

MJS is an investigator at the Faculty of Medicine and Dentistry at the University of Alberta. DF is a Senior Investigator at the Kaiser Permanent Northwest Center Health Research in Portland, Oregon, USA and a Professor *Emeritus *at the University of Alberta. David is a developer of Health Utilities Index Mark 2 and Mark 3 multi-attribute systems. David has a proprietary interest in Health Utilities Incorporated. SG is a biostatistician with especial interest in clinical trials. SG works at the Cross Cancer Institute in Alberta. RGN is an assistant professor at the Faculty of Medicine and Dentistry at the University of Alberta. JW is an associate professor at the Faculty of Medicine and Dentistry at the University of Alberta. KJ is the senior transplant coordinator and is in charge of the lung transplant database. MS is a transplant coordinator. DZ is the team pharmacist. GH is the dietician for heart and lung transplant teams. DL is the director of the lung transplant program and professor at the Faculty of Medicine and Dentistry at the University of Alberta.
